# Analysis of the coding potential of the partially overlapping 3' ORF in segment 5 of the plant fijiviruses

**DOI:** 10.1186/1743-422X-6-32

**Published:** 2009-03-17

**Authors:** Andrew E Firth, John F Atkins

**Affiliations:** 1 BioSciences Institute, University College Cork, Cork, Ireland; 2 Department of Human Genetics, University of Utah, Salt Lake City, UT 84112-5330, USA

## Abstract

The plant-infecting members of the genus *Fijivirus *(family *Reoviridae*) have linear dsRNA genomes divided into 10 segments, two of which contain two substantial and non-overlapping ORFs, while the remaining eight are apparently monocistronic. However, one of these – namely segment 5 – contains a second long ORF (~200+ codons) that overlaps the 3' end of the major ORF (~920–940 codons) in the +1 reading frame. In this report, we use bioinformatic techniques to analyze the pattern of base variations across an alignment of fijivirus segment 5 sequences, and show that this 3' ORF has a strong coding signature. Possible translation mechanisms for this unusually positioned ORF are discussed.

## Findings

The genus *Fijivirus *is one of ≥12 genera within the *Reoviridae*, a family of segmented dsRNA viruses. Fijiviruses have 10 segments, and infect plants and insects. Species such as Fiji disease virus (FDV), Mal de Rio Cuarto virus (MRCV) and Rice black streaked dwarf virus (RBSDV) are transmitted by planthoppers and replicate in both the insect and plant hosts, while the more distantly related *Nilaparvata lugens reovirus *replicates only in insects. Reovirus segments are predominantly monocistronic. However, two of the plant fijivirus segments (S7 and S9 in RBSDV, homologous segments in other sequenced plant fijiviruses) contain two non-overlapping coding sequences (CDSs), each pair separated by a short non-coding sequence [1,2]. One other plant fijivirus segment (S5 in RBSDV) contains a second substantial open reading frame or ORF (hereafter ORF5-2; ~200+ codons), that overlaps the 3' end of the 'major' CDS (hereafter ORF5-1; ~920–940 codons) in the +1 reading frame. The presence of this open reading frame has been noted previously in RBSDV [3,4]. However, it has often been ignored in the fijivirus literature; it is not currently annotated in any of the three GenBank plant fijivirus RefSeqs; and its unusual genomic location means that its coding status remains uncertain without further analysis. In this short report, we present bioinformatic evidence that ORF5-2 is in fact coding, and initiates ≥365 nt before the 3' end of ORF5-1 – thus implying that it utilizes an unusual, as yet undefined, expression mechanism.

Fijivirus sequences in GenBank (9 Feb 2009) homologous to RBSDV segment 5 were located by applying tblastn [5] to the RBSDV segment 5 RefSeq [GenBank: NC_003736] ORF5-1 amino acid sequence, resulting in the additional sequences [GenBank: AY144569] – RBSDV segment 5, [GenBank: NC_007160] – FDV segment 5, and [GenBank: NC_008735] – MRCV segment 5. Note that the GenBank RefSeqs NC_003736, NC_007160 and NC_008735 were derived, respectively, from AJ409147, AY029521 and AY607587.

If initiation is assumed to occur at the first in-frame AUG codon then, in the RBSDV RefSeq [3], the two segment 5 ORFs have nucleotide coordinates 16–2826 (ORF5-1; 107 kDa) and 2378–3070 (ORF5-2; 26.7 kDa), so that ORF5-2 has 231 codons and overlaps the 3' end of ORF5-1 by 449 nt in the +1 reading frame. In the other RBSDV sequence (AY144569; [4]), ORF5-1 has the same nucleotide coordinates (i.e. 16–2826), but the first suitable AUG codon for ORF5-2 is 28 codons further 3', giving ORF5-2 the maximal AUG-initiated nucleotide coordinates 2462–3070 (203 codons; 23.4 kDa; 365 nt overlap). In MRCV [6], the maximal AUG-initiated nucleotide coordinates are 16–2811 (ORF5-1) and 2366–3130 (ORF5-2), so that ORF5-2 has 255 codons and overlaps the 3' end of ORF5-1 by 446 nt, again in the +1 reading frame.

In the FDV RefSeq, NC_007160, the two overlapping ORFs have apparently been 'merged' into a single long ORF [7]. However, in the expected overlap region, there is a +1 frame overlapping ORF of up to 128 codons that has high amino acid homology to the N-terminal half of ORF5-2 in RBSDV and MRCV. Moreover, the C-terminal ~106 amino acid region of the annotated FDV ORF5-1 has significant homology to the C-terminal half of ORF5-2 in RBSDV and MRCV. This suggests that NC_007160 contains a deletion within or near to the region 2718–2746. Whether this is a real feature of FDV, or whether NC_007160 simply represents a 'defective' sequence, merits further investigation. For the analyses presented here, however, an additional 'U' was arbitrarily inserted into a run of five 'U's at the location 2714–2718 and, in this case, ORF5-1 and ORF5-2 assume respective maximal AUG-initiated nucleotide coordinates 59–2827 and 2448–3065, so that ORF5-2 has 206 codons and overlaps the 3' end of ORF5-1 by 380 nt in the +1 reading frame. (Note that our results do not depend on the FDV sequence – if it is excluded from the analyses, we obtain essentially the same results for the remaining fijivirus sequences.)

**Figure 1:**
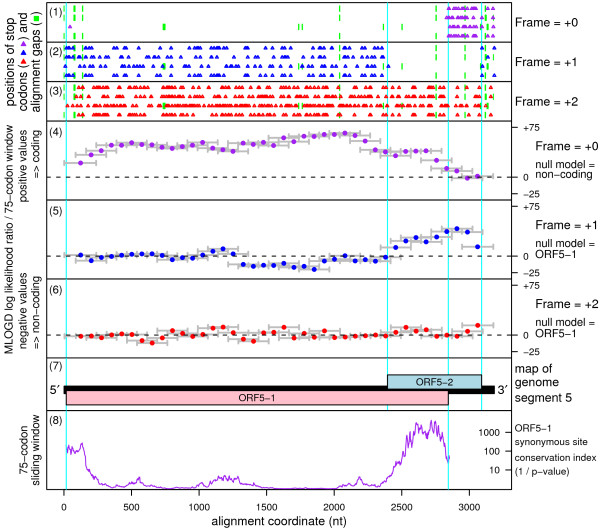
**Coding potential statistics for an alignment of four plant fijivirus segment 5 sequences**. **(1)-(3) **The positions of stop codons in each of the four sequences in each of the three forward reading frames (frame defined by alignment to the reference sequence [GenBank: NC_003736]). The FDV sequence has been put into the same frame as the other sequences via the arbitrary insertion of an extra 'U' into the run of five 'U's at NC_007160 nucleotides 2714–2718, as discussed in the text. Note the conserved absence of stop codons in the +0 frame within ORF5-1 and in the +1 frame within ORF5-2. **(4)-(6) **MLOGD sliding-window plots (window size 75 codons; step size 25 codons). Each window is represented by a small circle (showing the likelihood ratio score for that window), and grey bars showing the width (ends) of the window. See [9] for further details of the MLOGD software. In **(4) **the null model, in each window, is that the sequence is non-coding, while the alternative model is that the sequence is coding in the +0 (i.e. ORF5-1) frame. Positive scores favour the alternative model and, as expected, there is a strong coding signature throughout ORF5-1. In **(5)-(6) **the null model, in each window, is that only ORF5-1 is coding, while the alternative model is that both ORF5-1 and the window frame are coding. Scores are generally negative with some scatter into low positive scores, except for the ORF5-2 region which has consecutive high-positively scoring windows **(5)**. Note that the generally lower MLOGD signal within the overlap region itself **(4)-(5)**, and also at the 5' end of ORF5-1 **(4)**, is due to there being fewer substitutions with which to discrimate the null model from the alternative model in these regions of above-average nucleotide conservation. **(7) **Map of the reference sequence [GenBank: NC_003736]. **(8) **Conservation at synonymous sites within ORF5-1 (see text and [18] for details). Note that the relatively large window size (75 codons) – used here for improved statistical power – explains the broad smoothing of the conservation peak at the edges of the region where ORF5-2 and ORF5-1 overlap.

Overlapping CDSs are difficult to analyze with conventional gene-finding software [8]. The software package MLOGD, however, was designed specifically for identifying and analyzing such CDSs, and includes explicit models for sequence evolution in multiply-coding regions [8,9]. Using MLOGD, we recently identified – and subsequently experimentally verified – a new short CDS in the *Potyviridae *family that overlaps the P3 cistron but is translated in the +2 reading frame [10]. When we applied MLOGD to an alignment of the four fijivirus sequences, MLOGD detected a strong coding signature for ORF5-2 (Figure 1). The number of independent base variations across the alignment within the ORF5-2 region is N_var _~ 484, and the total MLOGD score is log(LR) ~88.9 (see [9] for details). Extensive tests with known single-coding and double-coding virus sequence alignments indicate that 'N_var _≥ 20' and 'log(LR) ≥ $\frac{1}{6}$ × N_var_' signals robust detection (<1% false positive rate) of an overlapping same-strand CDS [9] (and unpublished data).

The bottom panel of Figure 1 shows a representation of the conservation at synonymous sites in ORF5-1. Beginning with pairwise sequence comparisons, conservation at synonymous sites (only) was evaluated by comparing the observed number of base substitutions with the number expected under a neutral evolution model. The procedure takes into account whether synonymous site codons are 1-, 2-, 3-, 4- or 6-fold degenerate and the differing probabilities of transitions and transversions (full details are available on request from the authors). Statistics were then summed over a phylogenetic tree as described in [9], and averaged over a sliding window. Peaks in the conservation at synonymous sites are generally indicative of functionally important overlapping elements – including overlapping CDSs – and it can be seen that the highest conservation at synonymous sites in ORF5-1 corresponds to the region where it overlaps ORF5-2.

Further, albeit indirect, evidence for the coding status of ORF5-2 comes from an analysis of the 3'UTR lengths of the 10 segments. In RBSDV, for example, the 3'UTR lengths for segments 1–4 and 6–10 are 71, 86, 117, 74, 185, 81, 136, 111 and 103 nt. If ORF5-2 is not a CDS, then segment 5 has an unusually long 3'UTR (335 nt). However, if ORF5-2 is coding, then the 3'UTR length (91 nt) is within the range of 3' UTR lengths for the other 9 segments.

Since subgenomic RNAs are unknown in the *Reoviridae *family, the genomic location of ORF5-2 rules out most possible translation mechanisms. In NC_003736 (RBSDV), for example, ORF5-1 potentially begins at AUG1 while ORF5-2 (if AUG-initiated) begins at AUG56 or later, thus precluding conventional leaky scanning [11]. Reinitiation appears unlikely since, given the 5'-extent of the positive MLOGD coding signal, it would appear to necessitate backward scanning of ≥365 nt (cf. [12]). Transcriptional slippage also appears unlikely as no suitable slippage sites were found and, in contrast to the paramyxovirus 'rule of six' [13], there is no obvious mechanism in the reoviruses for selective packaging of non-edited transcripts. Ribosomal +1 frameshifting from ORF5-1 into ORF5-2 to produce a fusion protein (in competition with conventional translation of ORF5-1) is one possibility [14]. A second possible mechanism is an IRES – examples of which in other viruses can range from complex RNA secondary structures (which we were not able to identify in fijivirus segment 5, in any convincing manner, with the available sequence data) to simple polypurine tracts [15,16]. A third possible translation mechanism is some sort of ribosomal shunting. Indeed in another *Reoviridae *species – *Avian reovirus *– a novel, as yet not fully understood, scanning-independent ribosome migration mechanism is used to bypass two upstream CDSs in order to translate the 3'-proximal CDS on the tricistronic S1 mRNA [17].

**Figure 2:**
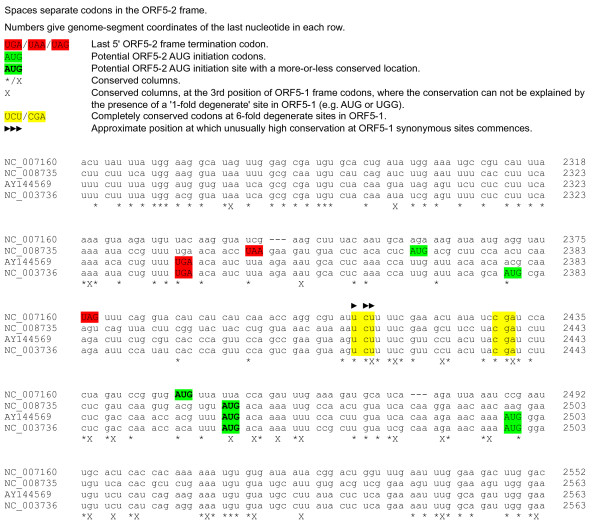
**Alignment extract showing the region around the 5' end of fijivirus ORF5-2**. The high nucleotide conservation in row 1 (mostly '*'s, only 2 'X's) can potentially be a result of amino acid constraints on the protein encoded by ORF5-1. In contrast, the high nucleotide conservation from the middle of row 3 to the end of the alignment extract is indicative of overlapping features (many 'X's).

A more detailed examination of sequence conservation around the 5' end of ORF5-2 is shown in Figure 2. If ORF5-2 is AUG-initiated, then the most plausible site would appear to be at NC_003736 coordinates 2462–2464, at which point all four sequences have a nearly-aligning AUG codon. However, the Kozak contexts of these AUG codons are relatively poor. Furthermore, the enhanced conservation at synonymous sites in the ORF5-1 reading frame commences around 14 codons further 5', including the completely conserved motif 'U CUU UUC G' (ORF5-2 frame codons; or 'UCU UUU CG' in the ORF5-1 frame) at NC_003736 coordinates 2419–2426. In particular, there are two completely conserved ORF5-1 frame codons (UCU/Ser and CGA/Arg) at six-fold degenerate sites at NC_003736 coordinates 2419–2421 and 2437–2439. One possibility is that these conserved motifs are involved in shunting, reinitiation or IRES activity to allow ORF5-2 initiation at the downstream conserved AUG codon. An alternative possibility is that these motifs mediate +1 frameshifting from ORF5-1 into ORF5-2 so that ORF5-2 is translated as part of a fusion product (~117 kDa in RBSDV).

Although much remains to be discovered about even the known fijivirus proteins, it is important to be aware of the full complement of encoded proteins as early as possible. We hope that presentation of this bioinformatic analysis will help fullfil that goal. Initial verification of ORF5-2 product could be by means of immunoblotting with ORF5-2 specific antibodies, bearing in mind, however, that it may be expressed at relatively low levels.

## Competing interests

The authors declare that they have no competing interests.

## Authors' contributions

AEF carried out the bioinformatic analysis and wrote the manuscript. Both authors edited and approved the final manuscript.
